# Ethical challenges in sensitive research: a reflective narrative on managing the clinician-researcher dual role

**DOI:** 10.1186/s12904-025-01850-y

**Published:** 2025-07-19

**Authors:** Yakubu Salifu

**Affiliations:** International Observatory on End of Life Care, Division of Health Research, Faculty of Health and Medicine, Lancaster University, Lancaster, UK

**Keywords:** Clinician-researcher, Cultural sensitivities, Dual role, Ethical dilemmas, Prostate cancer, Qualitative research, Reflexivity, Research ethics, Sensitive research, Therapeutic relationship

## Abstract

**Background:**

Conducting sensitive research in healthcare settings often presents complex ethical challenges, particularly when clinicians assume a dual role as researchers. This dual role, while providing valuable insight, can lead to ethical dilemmas when professional responsibilities intersect with research obligations, especially in resource-limited and culturally sensitive environments. This reflective narrative explores these ethical tensions through the lens of a qualitative study involving men living with advanced prostate cancer and their family caregivers.

**Method:**

The study employed a qualitative design, drawing on individual and dyadic interviews, as well as focus group discussions with healthcare professionals. A reflective narrative approach was chosen as the method to critically engage with the researcher’s personal experiences, ethical dilemmas, and the emotional complexities of conducting research in this context. The approach was justified as it provides a means of exploring both the data and the reflective, situated nature of the clinician–researcher role, which is essential for understanding the broader implications of conducting sensitive research.

**Results:**

The analysis revealed several key ethical challenges, including emotional labour, professional role conflicts, and the management of therapeutic boundaries. The clinician–researcher dual role significantly shaped participant consent, data collection, and interpretation of findings. It highlighted the vulnerability of both participants and researchers, especially in emotionally charged, culturally sensitive contexts. Ethical tensions often arose between maintaining professional objectivity and addressing the immediate care needs of participants, underscoring the importance of reflexivity in navigating these complexities.

**Conclusion:**

This reflection calls for enhanced ethical preparation and emotional resilience among nurse researchers, particularly those working with vulnerable populations in sensitive research areas like palliative care. The study emphasises the need for clear boundary protocols, structured reflexive journaling, and regular peer debriefing to help clinician–researchers navigate their dual roles while maintaining ethical integrity. The findings contribute to the discourse on ethical praxis in qualitative health research, offering practical strategies for managing the inherent challenges of conducting research in ethically complex environments.

**Supplementary Information:**

The online version contains supplementary material available at 10.1186/s12904-025-01850-y.

## What this article addresses

This reflective piece is crucial for understanding the ethical and emotional challenges faced by clinician–researchers, particularly in sensitive research with vulnerable populations in resource-limited settings. It adds valuable insights to the literature, highlighting global south-specific considerations and offering practical strategies for navigating the clinician-researcher dual role in palliative care, with implications for nursing practice, education, and policy development.

**Supplementary Information:**

The online version contains supplementary material available at 10.1186/s12904-025-01850-y.

## Introduction/Background

Research involving seriously ill participants often unfolds within emotionally charged terrain, but for clinician–researchers, this terrain carries an added ethical complexity: navigating a dual role. In palliative and end-of-life care research, where vulnerability, suffering, and uncertainty prevail, clinician–researchers may find themselves navigating tensions between their professional obligations to intervene and the methodological imperative to maintain critical reflexivity and ethical integrity [1, 2]. This dual positioning can generate ethical tensions around consent, therapeutic boundaries, and emotional involvement, particularly when clinical needs arise during the course of data collection.

Existing literature highlights the challenges of maintaining role clarity in such situations, especially when the researcher is also a nurse or healthcare provider [3, 4]. While the clinician–researcher’s identity can foster rapport and facilitate access to participants, it may also blur boundaries and complicate decisions about when—and whether—to intervene. These dilemmas become even more pronounced in resource-constrained or culturally sensitive settings, where healthcare gaps can amplify participants’ needs and clinician–researchers are often viewed as trusted authorities or caregivers.

A scoping of the literature identifies three core ethical challenges in sensitive research. First, terminally ill patients often face a significant burden and may experience research fatigue or a fluctuating capacity to provide informed consent, raising questions about the validity and ethics of their participation [5, 6]. Second, clinician–researchers frequently encounter distress and boundary tensions when their dual roles compel them to choose between intervening to relieve participant suffering and maintaining the integrity of the study—an equilibrium that can be difficult to sustain [7–10]. Third, cultural and contextual factors—such as social taboos surrounding death, disease-related stigma, and resource constraints—can profoundly influence both the conduct of data collection and participants’ willingness to share openly. Of these, the most central to this reflection is the clinician–researchers frequently encounter distress and boundary tensions when their dual roles compel them to choose between intervening to relieve participant suffering and maintaining the integrity of the study—an equilibrium that can be difficult to sustain [7–10]. In contrast, the first and third concerns—namely, the burdens of participation on terminally ill patients [5, 6] and the influence of cultural factors on disclosure [3]—though important, are treated here only in relation to how they intersect with the clinician–researcher dilemma.

Sensitive qualitative research with vulnerable populations—such as men living with advanced prostate cancer—inevitably raises complex emotional, ethical, and cultural challenges [11–13]. Emotional tensions arise when participants’ distress risks overwhelming the researcher’s composure, demanding skilled emotional labour. Clinician–researcher dual role dilemmas surface when professional obligations to act conflict with research neutrality, requiring careful ethical navigation. Cultural sensitivities, particularly around stigma, taboos, and collective decision-making, demand culturally competent, flexible engagement to foster trust and authenticity [14–18]. Dyad interviews further reveal the influence of collective meaning-making and necessitate sensitivity to culturally embedded reticence, particularly regarding death and illness disclosure. These issues frame the ethical context in which this study was conducted and underscore the need for heightened reflexivity and moral preparedness among clinician–researchers.

Drawing on a Ghanaian study of home-based prostate cancer care [13, 19, 20], this reflective narrative focuses on the practical and emotional demands of the clinician–researcher dual role. It addresses a significant gap in the literature: how to ethically respond when urgent clinical concerns arise during fieldwork. An illustrative case involving unsafe wound-dressing practices is used to interrogate the choice to observe, document, or intervene—and how to do so ethically and reflexively. This reflection proposes a practice-oriented framework, including boundary guidelines, structured journaling, and institutional peer support, to help clinician–researchers maintain ethical integrity while conducting research in sensitive, resource-limited settings.

### Ethics approval and consent to participate

This study was conducted in accordance with the ethical standards set out in the Declaration of Helsinki and relevant national and institutional guidelines for research involving human participants [21]. Ethical approval for this study was obtained from multiple bodies. Initial approval was granted by the Faculty of Medicine and Health Sciences (FMHS), University of Nottingham Ethics Committee (Ethics Reference Number: F12092016). Subsequently, a Certificate of Registration (Registration Number: RD/CR16/247) was secured from the Research Development Unit (RDU) of Komfo Anokye Teaching Hospital (KATH), where part of the fieldwork was conducted. This certificate, together with the research proposal, was submitted to the Committee on Human Research, Publications and Ethics (CHRPE) at the Kwame Nkrumah University of Science and Technology (KNUST)—the institutional affiliate of KATH—for local ethics approval (Ethics Reference Number: CHRPE/AP/496/16). In addition, prior to commencing fieldwork, permission was obtained from relevant hospital directorates where patient participants were to be recruited. Informed consent was obtained from all participants prior to their involvement in the study. Participants were fully briefed about the purpose of the research, the voluntary nature of their participation, and their right to withdraw at any time without consequence.

## Method

This article adopts a reflective narrative approach [22], informed by a constructivist qualitative paradigm, to critically explore the ethical challenges encountered during fieldwork with men living with advanced prostate cancer and their family caregivers in Ghana [23]. Rather than presenting the findings of the broader study in full, this paper focuses on the researcher’s experience of managing the dual role of clinician and researcher within a sensitive research setting. The reflective narrative centres on fieldwork encounters that exposed ethical tensions—particularly those related to emotional labour, therapeutic boundaries, and role conflict—and draws on field diaries, reflexive memos, and real-time decision-making moments to illuminate these challenges. Dyadic interviews were used to explore shared experiences between patients and caregivers. This method captured relational dynamics and co-constructed narratives but also posed challenges around disclosure and emotional protection [24–26]. These dynamics shaped the ethical dilemmas described in later sections.

### Context

The broader study, from which this reflection draws, explored the experiences of men with advanced prostate cancer, their caregivers, and healthcare professionals in a resource-poor Ghanaian setting. It employed semi-structured interviews and focus groups to investigate caregiving dynamics, home-based care, and cultural stigma. While these findings are reported elsewhere [12, 13, 19, 20, 27], the current article is focused solely on the ethical and emotional complexities encountered by the clinician–researcher during field engagement in that study.

### Reflexive process and data sources

Reflexivity formed the core of the analytical process. Following each interview or focus group, I recorded detailed field notes capturing both verbal content and non-verbal cues. These were supplemented by a field diary and reflexive memos, in which I documented emotional reactions, ethical uncertainties, and internal deliberations about how to navigate emerging clinical concerns. These reflections were analysed inductively, not to generate formal themes for reporting, but to trace patterns in how dual-role tensions emerged and were managed over time.

This reflective material was not treated as separate from the field experience, but rather as co-constructed data—an account of how meaning was shaped not just by participants’ stories, but by the ethical and emotional terrain navigated by the researcher. Regular debriefing with peers and mentors provided a space for externalising these reflections, allowing deeper ethical analysis and mitigating the risk of over-identification with participants [23].

#### Setting

This study was conducted in a resource-poor context, where access to healthcare facilities and support services was limited. The setting included both healthcare and community environments where men with advanced prostate cancer and their family caregivers were located.

#### Population and sample

The study focused on men living with advanced prostate cancer and their family caregivers in a resource-limited context. Participants were selected based on their involvement in palliative care settings, where they had direct experience with the challenges of living with advanced cancer. The sample included both patients and caregivers to explore the emotional and practical dynamics within these relationships.

#### Recruitment

Participants were recruited through local healthcare providers and support networks. Given the resource constraints, recruitment was purposeful, aiming to include those who were experiencing the complexities of advanced illness within this context. The recruitment process ensured that both patients and caregivers were willing to share their experiences and reflections on the challenges they faced.

#### Data collection

Data were collected using a reflective narrative approach, which allowed for the co-construction of meanings between the researcher and participants during the fieldwork. Individual and dyadic interviews were conducted with men living with advanced prostate cancer and their caregivers. Additionally, regular focus group discussions with healthcare professionals provided further insights into the broader healthcare environment. In each interview, I actively noted practical hurdles such as poor transport and cultural taboos that influenced participant engagement. Following each interview, I recorded my personal reflections in a field diary and reflexive memos to capture both the participants’ narratives and my own reactions. These notes were made immediately after each interview and during critical incidents, ensuring that real-time reflections were included in the analysis. Pseudonyms were assigned to all participants in this study to safeguard their anonymity and uphold their dignity.

The semi-structured interview guide developed specifically for this study to explore the experiences of men living with advanced prostate cancer in a resource-poor setting. The guide was informed by a review of the literature and tailored to the cultural and contextual dynamics of the study population. An English-language version of the interview guide and an abridged version of a distress protocol is provided as “Supplementary file’’.

### Positionality and reflexivity

Positionality and reflexivity are essential in qualitative research as they acknowledge how the researcher’s background, experiences, and perspectives influence every stage of the inquiry—from framing the research question to interpreting findings—ensuring transparency, ethical integrity, and deeper insight into the co-construction of meaning [28–30]. As a practising nurse and nursing academic, I entered this research with a dual professional identity that profoundly shaped the entire inquiry. My prior clinical experience as a nurse, inevitably influenced how I understood and emotionally responded to participants’ narratives of suffering, stigma, and resilience. These professional lenses did not merely accompany the research—they actively shaped the way I listened, interpreted, and engaged in the field.

Conducting the study in Ghana, my home country, positioned me in a complex insider–outsider role. Sharing language, cultural norms, and familiarity with the healthcare system helped build rapport and trust. However, this proximity also generated unspoken expectations—both from participants and within myself—that I might intervene, particularly when unmet clinical needs surfaced. Participants were informed during the consent process that I was a nurse by background but not acting in a clinical capacity. While this transparency appeared to support openness and disclosure, it also heightened my ethical and emotional entanglement with the field. Moments of suffering were not abstract or distant—they were situated within a community, a health system, and a culture I was deeply part of.

These intersecting identities—Black African, Ghanaian, nurse, researcher, and academic—placed me within and across multiple social and professional spaces. In qualitative research, the researcher is never a neutral observer, and when one wears the clinician’s “hat”, ethical complexities become intensified. I was acutely aware of how my positionality could shape not only participant responses but also the meaning I ascribed to those responses. To maintain ethical rigour and enhance trustworthiness, I engaged in continual reflexivity—not as a procedural task but as a foundational mode of being in the research.

A particularly sensitive dynamic emerged during focus group discussions with fellow nurses about their experiences of caring for men with advanced prostate cancer as part of the larger study. My role as a university lecturer and as senior to some of those nurses may have introduced a perceived authority, potentially influencing how participants responded or self-censored. Some participants may have worried about judgement, while others might have felt more comfortable sharing due to our shared professional background. These shifting dynamics demanded constant attention to how my presence, tone, and body language influenced the group. Strategies such as explicitly affirming the non-evaluative nature of the study, adopting a posture of humility, and actively listening were essential to minimising power imbalances and fostering authentic dialogue [31].

Reflexivity was embedded in all stages of the research. A field diary served as a vital tool—not only to document observations, but to critically examine my reactions, assumptions, and emotional responses. After each interaction, I recorded detailed reflections that included both verbal exchanges and non-verbal cues. This process enabled me to attend closely to what participants said, how they said it, and what remained unsaid. Rather than applying a fixed analytic frame, I allowed themes to emerge from this interplay between narrative and reflection, aligning with the constructivist view that knowledge is co-created between researcher and participant.

This ongoing reflexive practice sharpened my awareness of when I was at risk of slipping into a clinical role or allowing empathy to override methodological clarity. It helped me to sit with discomfort, to resist the impulse to act, and instead to document the ethical tensions that arose. Far from weakening the research, these moments of uncertainty and moral ambiguity became central to the reflective analysis.

Through acknowledging and engaging with my positionality, I situate this narrative within the lived, relational, and emotionally layered context in which it occurred. Reflexivity was not an afterthought but an ethical orientation that informed how I engaged with participants, how I handled their stories, and how I represented their realities. It is this transparency and critical self-awareness that lends credibility to the reflective insights presented here and positions this piece not simply as a recounting of fieldwork, but as an ethically engaged and methodologically robust contribution to palliative care research.

### Data analysis

Data analysis was conducted through an iterative process. Field notes, diary entries, and interview transcripts were systematically reviewed and analysed together to facilitate an ongoing dialogue between emerging findings and reflective insights. Reflective narrative was central to the data analysis process, providing a means to explore the dynamic relationship between the researcher and participants [23]. As a nurse-researcher, my dual role influenced both the data collection and analysis, making reflexivity a crucial element in interpreting the findings. After each interview, I recorded immediate reflections, emotional responses, and contextual observations in a field diary. This reflective journaling allowed me to capture not only the participants’ words but also my reactions to their experiences, which were instrumental in understanding the emotional and ethical complexities of the research.

During data analysis, these reflective entries were analysed alongside the interview transcripts and field notes. The iterative approach ensured that the data was reviewed multiple times, with emerging themes identified through open coding [32, 33]. Reflexivity allowed me to recognise how my professional background and personal experiences shaped the interpretation of the data, helping me to remain aware of potential biases and over-identification with participants’ stories. The use of reflective narrative facilitated a deeper understanding of the emotional and social dynamics of caregiving and living with advanced prostate cancer. It enabled a co-construction of meaning between the researcher and participants, enriching the analysis with nuanced insights and ensuring the integrity of the findings. Themes were developed inductively through open coding, while critically examining how my positionality as a nurse-researcher shaped the interpretation of the data [33]. Regular debriefings provided reflexive dialogue, helping me remain aware of my positionality and prevent over-identification with participants’ experiences.

### Emotional and ethical tensions in sensitive research

This vignette illustrates one of the most emotionally complex dilemmas I encountered during an interview. During an interview with Mr. Ofori, 67, a participant with advanced prostate cancer, we sat on low wooden stools beneath a veranda as his wife hovered nearby, folding laundry but listening quietly. He spoke in a low, laboured voice, pausing often to catch his breath, his hand trembling slightly as he pointed to a half-empty box of pills on the table. When he spoke of being unable to afford more pain medication, tears filled his eyes—and his wife turned away, wiping her face on the corner of her headscarf. The silence that followed felt heavy, and although I wanted to comfort them, I remained still, aware that my reaction carried ethical weight. This moment confronted me with a sharp ethical dilemma: beyond listening, was there more I should do?

As a nurse, I felt the moral pull to alleviate their suffering—perhaps to offer direct financial assistance or help secure medications—but I also understood the risk of overstepping the researcher boundary and undermining voluntary consent through unintended coercion. I recall considering whether my presence might raise expectations I could not fulfil. I chose not to intervene financially at that moment but noted the need to raise this with the palliative care team during my debriefing.

Participants in this study were given modest compensation to cover travel costs or time, as approved by the ethics committee. However, situations like this revealed how limited such compensation could feel in the face of genuine material suffering. This experience raised questions about exploitation and power: was I benefiting academically from the pain of those whose immediate needs I could not meet? Balancing empathy with the need to collect data prompted an internal conflict. Such ethical dilemmas are not uncommon in qualitative research, particularly when dealing with vulnerable populations [26, 34]. The tension between respecting the dignity of participants while capturing their authentic, often painful, narratives is a critical consideration in sensitive research [35–37].

### Clinician-researcher dual role dilemmas

Another ethical challenge arose when a participant shared, through tears, the physical and emotional pain he was experiencing during the interview. The following account highlights a recurring dilemma in navigating clinician-researcher dual role.

The interview took place in a modest sitting room with cracked windows and faded family portraits on the wall. The participant, a man in his seventies, sat upright in a plastic chair, but his breathing was shallow, and his shirt clung to his back with sweat. As we began speaking, he winced repeatedly and shifted uncomfortably. Within minutes, it was clear he was in escalating pain. His sentences trailed off, and he closed his eyes, gripping the armrest with increasing tension. I paused and asked if he wished to stop, to which he gave a faint nod. In that moment, I was no longer sure if I was acting as a researcher or silently slipping into my clinical instincts. As the interview unfolded, Mr. Boat disclosed persistent discomfort and fever. When asked casually about wound care, it became evident that the suprapubic catheter site—a key part of his palliative care—was inflamed and being cleaned using a reused flannel cloth due to lack of sterile supplies. The smell from the wound was noticeable, and visible signs of infection, including redness and pus, raised serious concerns for sepsis risk.

The dilemma here was about ‘*To Disclose or Not Disclose*?’ [38]. As a researcher, I was there to listen, collect narratives, and avoid clinical intervention to preserve the integrity of the research setting. However, as a nurse, my professional code mandated that I act to prevent harm [39]. Caught between the roles of a researcher and clinician, I experienced profound emotional tension: sadness at the systemic neglect Mr. Boat faced, guilt for potentially remaining silent, and anxiety about compromising the research’s ethical framework. After a moment of internal deliberation, I carefully negotiated the boundary. I paused the formal interview, sought consent from the family, and gently explained the infection risks. I offered to contact the palliative care team at the nearby hospital for urgent follow-up rather than directly providing treatment myself. Being a clinician–researcher bridges the gap between practice and evidence, ensuring that research remains grounded in real-world clinical experience. When a researcher–clinician observes something during the study—such as a symptom or condition that poses a health risk—they are ethically obliged to act in the patient’s best interest by facilitating appropriate disclosure and referral, aligning with professional standards of care [40].

This carefully calibrated intervention preserved my role as a researcher while fulfilling a basic ethical duty to safeguard participant wellbeing [41]. Reflecting afterwards, I realised how emotional labour—managing distressing observations, navigating personal sadness, and maintaining professional composure—forms a hidden but critical part of sensitive qualitative research. It highlighted the real-world complexity of balancing research objectivity with human compassion.

Building trust beforehand and securing iterative consent enabled a respectful response. This incident underlines the importance of training for nurse-researchers in managing emotional and ethical tensions, and ensuring clear protocols are in place when participant safety becomes a concern corroborating previous work [42].

### Cultural sensitivities and stigma in research

Engaging with culturally embedded stigma around illness required careful ethical navigation as a clinician–researcher working within a shared but sensitive cultural context. In Ghana, prostate cancer carries not only medical but social implications—silence, shame, and secrecy often shaped how participants engaged with the research encounter, demanding heightened reflexivity around disclosure, trust, and relational boundaries [43]. As a clinician–researcher, navigating cultural stigma around prostate cancer in Ghana raised complex ethical and relational dilemmas. Several potential participants declined to be involved, voicing fears about community judgment and reputational harm if seen speaking openly about their illness. Their reluctance underscored the dual pressures I faced—not only to respect cultural silence, but also to encourage disclosure for the sake of research integrity. This tension shaped how I approached consent, phrased questions, and interpreted hesitations during interviews.

During interviews, participants often spoke hesitantly about sensitive issues such as urinary incontinence and sexual dysfunction—topics heavily stigmatised [15, 44, 45] because it is a shame-laden disease [18]. As a nurse familiar with these symptoms, I understood their clinical significance; yet as a researcher, I had to resist the urge to respond therapeutically. Trust-building became not just an ethical necessity but a methodological strategy, and I had to constantly reflect on how my clinical identity shaped participant expectations.

These dynamics challenged my assumptions about disclosure. I realised that trust could not be presumed even with shared cultural background—it had to be earned through non-judgmental listening and sustained affirmation of confidentiality. Participants’ silences taught me that meaning is often carried not only in what is said, but in what is withheld—a crucial insight for qualitative researchers operating in taboo-laden fields.

Ultimately, this experience not only enriched my understanding of the complex interplay between culture, stigma, and health communication but also contributes valuable insights to the growing body of global nursing research that advocates for decolonised, participant-centred methodologies. Culturally sensitive research is crucial not only for improving practice but also for dismantling the inequities ingrained in global health systems. As some researchers argue, embracing a decolonised approach means recognising and valuing local knowledge, which directly challenges the dominance of Western models in healthcare research [46, 47]. In nursing, this approach empowers marginalised communities, ensuring that research is not just reflective of diverse needs, but actively contributes to a more equitable, inclusive healthcare system.

### Dyad interviews and death taboos

Conducting dyadic interviews within a cultural context where open discussion of death is often taboo introduced unique ethical and emotional complexities. While these interviews offered rich insight into relational care dynamics, they also surfaced tensions around what could be said, by whom, and in whose presence—especially when cultural norms dictated silence around dying and prognosis. Dyadic interviews capture the co-constructed narratives and relational dynamics between participants, yielding deeper insight into how a patient and a caregiver jointly navigate care responsibilities and shared experiences. However, the presence of both individuals can sometimes inhibit candid disclosure of sensitive or conflicting feelings, because the pair may self-censor to protect each other or preserve harmony [26]. Conducting a dyadic interview with Mr. Karikari and his wife highlighted the profound cultural sensitivities surrounding discussions of death in Ghana. When the topic of dying naturally surfaced, Mrs. Karikari h avoided the word entirely, instead referring to it obliquely as “what might happen one day.” This subtle evasion is deeply rooted in Ghanaian cultural beliefs, where speaking openly about death in the presence of a seriously ill person is often thought to hasten its occurrence. The silence surrounding death is protective, intended to shield both the individual and family from emotional and spiritual harm.

Recognising the discomfort, I consciously chose not to press the topic. Instead, I allowed the participants to steer the conversation, privileging their narrative rhythms over a rigid interview schedule. This approach is consistent with culturally safe research principles, which advocate for researcher flexibility, participant autonomy, and the prioritisation of relational ethics over procedural rigidity. This experience underscored that sensitivity to non-verbal cues—hesitations, glances, shifts in tone—was as critical as attending to verbal content. Participants’ meaning making was often embedded in what was left unsaid, in silences and euphemisms, reinforcing “unspeakable domains” of health experience. Such domains demand an interpretive openness that goes beyond conventional interviewing techniques.

Respecting cultural taboos during research is not merely about avoiding offense; it is about protecting psychological safety and maintaining trust. Forcing uncomfortable conversations risks traumatisation, withdrawal, or the erosion of participant dignity [48–50]. In nursing research, particularly in end-of-life care studies, this sensitivity is vital for eliciting authentic narratives and supporting ethical participant engagement. This encounter contributes to nursing practice and research by emphasising the necessity of culturally responsive methodologies, especially in palliative and oncology care. Nurses working in multicultural contexts must be trained not only in technical communication skills but also in cultural humility and the ethics of presence—being attuned to what patients and families can and cannot bear to articulate. In research, flexible, participant-led approaches ensure that findings genuinely reflect lived experiences rather than researcher-imposed narratives.

### Managing participant vulnerability and unexpected deterioration

Responding to participant deterioration during interviews blurred the line between clinical instinct and researcher detachment, raising urgent ethical questions. A follow-up visit with Mr. Gyasi, who had previously appeared stable (a few weeks ago), unfolded into a moment of visible vulnerability and escalating distress, requiring an immediate recalibration of my role and responsibilities. During a scheduled second home visit with Mr. Gyasi, an elderly interview participant, I was confronted with a stark shift in ethical responsibility. Upon arrival, it was immediately evident that his condition had deteriorated significantly; he appeared frail, disoriented, and unable to engage in conversation. Although I had intended to collect follow-up data, my research aim became secondary to the immediate imperative of safeguarding his wellbeing. I abandoned the interview, provided reassurance, and assisted in alerting his family to seek urgent care.

This experience reflects a critical ethical principle in palliative care research: the primacy of participant welfare over data acquisition. In palliative contexts, participants’ health status can fluctuate rapidly and unpredictably, demanding that researchers practice continuous ethical reflexivity rather than adhering rigidly to data collection plans [51]. Research ethics are not static checkpoints but living processes that must evolve responsively in real time. Abandoning the interview was not a research “failure”; rather, it was an ethical success. Maintaining the dignity, comfort, and autonomy of participants, particularly those at end-of-life, is fundamental to trustworthy and compassionate research. Ignoring Mr. Gyasi’s visible distress in favour of pursuing data would have constituted a profound ethical breach, potentially eroding trust not only with the individual but with the broader community from which participants were drawn.

This incident also reinforces an important methodological lesson for nursing research: researchers must be trained to recognise when to withdraw, when to prioritise care over inquiry, and when to act swiftly as patient advocates. Particularly in global and resource-limited settings, where healthcare access may be precarious, the researcher’s role can momentarily intersect with that of a first responder. In these moments, responsiveness becomes an ethical imperative, not a discretionary act. The contribution to nursing practice here is twofold. First, it underscores that palliative care research must incorporate structured flexibility and real-time ethical decision-making as core competencies. Second, it highlights the need for nursing education programs to prepare researchers and practitioners for ethically complex, emotionally charged situations, emphasising humanistic care over procedural adherence. Ultimately, this experience reaffirmed that in the delicate landscape of palliative research, ethical vigilance, compassion, and the ability to prioritise humanity over inquiry are the true measures of research excellence.

One particularly challenging interview involved a participant who began to visibly deteriorate mid-conversation. He was sweating profusely and struggling to maintain a seated posture. As a clinician, I recognised the signs of physical distress that warranted immediate attention. Yet, as a researcher, I was also aware of the risk of disrupting rapport or undermining the participant’s agency by prematurely halting the interview.

This moment demanded an ethical decision grounded in my clinical judgment and relational sensitivity. I paused the interview, offered water, and gently asked whether he wished to continue or take a break. His quiet nod to stop was a clear indication of distress, and I honoured that choice without hesitation. This situation highlighted the unpredictable and emotionally charged nature of interviewing seriously ill individuals and the need for clinician–researchers to prioritise patient dignity over data collection.

### Therapeutic relationship

The emotional depth of palliative care interviews can generate therapeutic moments that challenge the boundaries of the researcher role [52–54]. As participants shared fears, regrets, and existential questions, I found myself emotionally drawn into the space of comfort and connection—requiring constant awareness of when empathy shaded into informal counselling or emotional overreach. For example, midway through the interview, a caregiver began to cry as she described feeling abandoned by the healthcare system. She asked me, “What would you do if it were your father?“—clearly seeing me as both nurse and confidant. I felt the urge to comfort and advise her but had to consciously hold back, aware of the power and boundaries of my dual role. Qualitative interviews play a pivotal role in fostering researcher-participant connections, particularly in palliative care where compassion and relational engagement are vital [55, 56]. My fieldwork affirmed that home visits were interpreted by participants as profound acts of love and concern, providing not only cathartic relief but a rare sense of empowerment often missing in clinical settings. Stella, a caregiver, reflected on the absence of home-based care and welcomed my visit, aligning with WHO’s advocacy for community-integrated palliative care services. While it is recommended that nurse researchers must not assume therapeutic roles, in low-resource contexts like Ghana, the lines often blur. Participants valued the emotional connection, suggesting that empathetic engagement, even when detached from formal therapy, can help sustain dignity in life-limiting illness. However, such relational dynamics intensify the ethical responsibility to manage boundaries carefully.

Flexibility in interviewing methods was essential: individual interviews allowed private disclosure of sensitive information, whereas dyadic interviews illuminated shared experiences of illness, offering deep insight into family dynamics [26, 34]. In dyadic settings, caregivers carefully avoided references to death, reflecting Ghanaian cultural norms where discussing death is considered socially dangerous. The relational closeness that developed, with participants inviting me to funerals and contacting me with health updates, mirrors findings by other researchers on the porous boundaries between researcher and participant in palliative care research [57, 58]. Managing these complex emotional entanglements demands preparation, emotional resilience, and ongoing ethical reflexivity [51, 59, 60]. Consequently, nurse researchers must resist assuming that clinical experience alone suffices; specialised training for sensitive, end-of-life qualitative research remains essential.

## Discussion

This reflective narrative offers a grounded and emotionally engaged account of the ethical tensions experienced by a clinician–researcher conducting fieldwork in palliative and end-of-life care in Ghana. As a nurse embedded in the cultural and clinical realities of the research context, I was continually required to negotiate the boundary between being present as a researcher and acting from my training and moral orientation as a nurse. These tensions—while not unique to palliative care—are heightened in this field, where vulnerability, suffering, and the imminence of death infuse the research encounter with urgent ethical and emotional significance.

The dual-role dilemma described in this paper resonates with broader concerns in qualitative research about relational ethics, role confusion, and the embodied labour of researcher reflexivity [57, 58]. However, it also extends this conversation by showing how these dilemmas are shaped by cultural and structural conditions—such as healthcare resource scarcity, stigma around illness, and expectations of care from participants—especially when the researcher is seen as both insider and authority figure. These dynamics raise difficult questions: When does observation become neglect? How do we hold space for suffering without rushing to “fix” it? What does ethical research look like when one is positioned, even symbolically, as a healer?

Managing moments of visible participant deterioration or emotional collapse tested my capacity for ethical attentiveness. These were not just moments of decision-making, but of embodied reckoning—where the desire to help clashed with the obligation to respect autonomy and maintain methodological integrity [1, 61]. Responding with silence, withdrawal, or a break in the interview were not neutral actions; they were ethically weighted gestures. These moments demanded a kind of real-time ethical improvisation rooted in both clinical judgement and relational sensitivity.

Therapeutic relationships also emerged organically within interviews, as participants reached out emotionally, physically, or symbolically for comfort, guidance, or care [49, 50, 62–64]. While these connections were powerful, they also risked confusing roles and inflating expectations. Honouring participants’ trust without overstepping ethical boundaries required ongoing inner negotiation. As a clinician–researcher, I had to become comfortable with discomfort—learning to be present and compassionate without rescuing, fixing, or taking on a clinical role that could disrupt the trust placed in the research process.

The use of flexible and culturally responsive methods—such as dyadic interviews, pauses, or shifting between languages—was not merely pragmatic, but ethical. These adaptations honoured participants’ dignity and autonomy, especially in a cultural context where death and illness are enmeshed in collective, often silent, meaning making. Cultural humility required not only sensitivity to local norms and taboos, but a willingness to listen for what was not said, to respect silence as a form of truth, and to accept that my role was not to extract information, but to witness and reflect ethically. The discomfort participants showed when discussing death during dyadic interviews illustrates the need for culturally responsive interviewing and training in relational ethics. Research in palliative care must respect silence as an ethical mode of communication and incorporate flexible, participant-led methods.

These challenges are highly relevant to nursing practice and research: they underscore the need for reflexivity, emotional resilience, cultural humility, and ethical preparedness. For the nursing workforce, particularly in palliative care and global contexts, ethical competence in research is not optional but a fundamental professional duty. Recognising that knowledge is co-produced in intimate and often painful relational spaces, nurses must be equipped to navigate dual-role dilemmas not with fixed scripts, but with moral imagination, institutional support, and reflexive tools.

This reflection contributes to understanding how researchers can navigate ethical and emotional tensions in palliative care research by acknowledging the complexity of these challenges—including how gender, particularly norms around masculinity, can influence both the expression of distress and the receipt of care. Recognising these gendered dynamics is essential for conducting research that is sensitive to power, vulnerability, and the often-invisible emotional labour within caregiving relationships [65]. It emphasises the need for careful consideration of participant welfare, ensuring that data collection does not overshadow the dignity of those sharing deeply personal and distressing experiences. The ethical imperative to protect, respect, and care cannot be separated from the methodological choices we make.

This work also supports calls for integrating cultural humility and reflexivity into global nursing education [66–74], and for moving beyond cultural awareness to decolonised, participant-centred practice [75–78]. It adds to a growing body of literature that calls for research training to include not just technical or procedural ethics, but the emotional, relational, and context-specific ethics of fieldwork—particularly for clinician–researchers working in cross-cultural or resource-constrained settings.

This reflective narrative contributes a grounded, practice-based insight into the ethical landscape of palliative care research. It highlights the clinician–researcher as a moral actor whose presence in the field is both a methodological asset and an ethical responsibility. In doing so, it invites a more honest, situated, and compassionate dialogue about what it means to do research with—not just about—people who are living with suffering. It is only through embracing this complexity that we can build more ethically sound and humanising approaches to palliative care research.

### Implications for practice, future research, and contributions to knowledge

This narrative study underscores the often-overlooked ethical and emotional labour involved in navigating the dual role of clinician and researcher in palliative care settings. While ethical guidelines typically address consent, confidentiality, and risk mitigation, they rarely account for the lived complexities of emotional proximity, therapeutic expectation, and cultural entanglement. With careful reflection on moments of role conflict, moral discomfort, and boundary tension, this paper contributes new insight into how ethical dilemmas unfold in real time, and how clinician–researchers must develop skills not only in research ethics, but in reflexive, relational, and moral discernment.

These reflections carry important implications for nursing and palliative care research. First, research training programmes should move beyond procedural ethics to include narrative-based, situational ethics training, where clinician–researchers are encouraged to explore case-based dilemmas, emotional self-awareness, and boundary negotiation. Reflexivity, cultural humility, and role clarity should be treated as core competencies, particularly for those conducting sensitive research with vulnerable populations.

Second, institutions should recognise the emotional toll and ethical uncertainty that often accompany clinician–researcher fieldwork. Building formal support systems such as reflective supervision, ethics mentoring, and structured debriefing sessions could help mitigate moral distress and support researchers’ emotional wellbeing. These supports are especially critical for researchers working in under-resourced or culturally sensitive settings, where expectations to intervene may be heightened.

Third, this work challenges the research community to more critically engage with how positionality, power, and practice intersect in qualitative research. The clinician–researcher is not a methodological liability, but a moral and relational actor, whose embeddedness can enhance trust, access, and meaning-making—when navigated with care. The field must therefore embrace reflective narratives not as anecdotal or supplementary, but as central sources of ethical and methodological insight.

Finally, this study contributes to a growing body of literature calling for a decolonised, participant-centred approach to global health research, where knowledge is co-produced through humility, responsiveness, and mutual respect. The ethical tensions explored here—of silence, intervention, emotional presence, and role ambiguity—are not failures of design but integral aspects of human research. Recognising and addressing them strengthens both the rigour and compassion of palliative care inquiry.

### Strengths and limitations

A major strength of this study lies in its use of a reflective narrative approach, which enabled deep, contextualised insights into the ethical challenges encountered during the research. As a result, these experiences may serve as a useful guide for clinician–researchers in palliative care settings. Additionally, because this study is part of a larger project that explored the perspectives of patients, caregivers, and healthcare professionals, it offers a holistic view of patients’ narratives, thereby enriching the credibility and depth of the findings. The researcher’s reflexive engagement further enhanced the authenticity of the ethical dilemmas explored, offering a transparent account of the emotional labour and role conflicts inherent in dual clinician–researcher roles. This approach contributes meaningfully to ethical discourse in qualitative health research, particularly in under-researched, resource-constrained contexts.

However, the study has certain limitations. This paper draws on a reflexive narrative approach rooted in personal field experiences, which by nature is deeply subjective and situated. While this enhances the richness and authenticity of the ethical reflections, it also carries limitations related to selective memory, emotional framing, and retrospective sense-making. The events described are filtered through my own positionality as a nurse, academic, and Ghanaian clinician–researcher, and are therefore not intended to be representative of all clinician–researchers’ experiences.

The reflections presented here are context-specific, shaped by the sociocultural environment of the field site and my professional identity. While these insights may be transferable to similar settings, they should not be taken as universally generalisable. Rather, they are offered as catalysts for dialogue and ethical reflexivity within the broader nursing, palliative care, and qualitative research communities.

## Conclusion

This reflection has underscored the emotional and ethical complexities of conducting palliative care research in resource-poor settings, especially when undertaken by clinician–researchers. It critically explored six interwoven ethical dimensions—emotional labour, professional role conflict, therapeutic engagement, cultural taboos, methodological sensitivity, and participant vulnerability—offering rich insight into the practical realities of sensitive fieldwork. This work is particularly significant for nurse researchers, whose dual roles require them to continuously balance their ethical duty of care with the demands of rigorous, reflexive inquiry.

What makes this study unique is its reflexive narrative grounded in real-world experience, which illuminates the moral dilemmas that arise when research intersects with human suffering, cultural beliefs, and clinical need. This reflective narrative highlights the ethical and emotional complexity of conducting sensitive research as a clinician–researcher in palliative care settings. Drawing on field experiences with men living with advanced prostate cancer in Ghana, I have explored how dual-role tensions manifest in moments of clinical deterioration, emotional disclosure, and cultural silence. These tensions demand not neutrality, but reflexive awareness and ethical responsiveness—qualities grounded in the clinician’s embodied knowledge and moral accountability.

Rather than offering fixed rules, this article contributes to a more nuanced understanding of how ethical boundaries are lived, negotiated, and re-negotiated in practice. The reflections presented here underscore the importance of structured reflexivity, peer support, and boundary guidelines in equipping clinician–researchers to respond ethically without undermining research integrity.

For nursing and allied health professions engaged in global palliative care research, these insights affirm the need to embed cultural humility, emotional preparedness, and relational ethics into research training and supervision. This is especially vital when working in under-resourced settings where clinicians may be called upon to act even when their formal role is observational.

Future work could expand these reflections by gathering comparative narratives across contexts, disciplines, and research traditions. While the reflections in this article are shaped by my positionality and the specific cultural setting of Ghana, they aim to provoke broader discussion about ethical preparedness and integrity in clinician-led qualitative research.

## Electronic supplementary material

Below is the link to the electronic supplementary material.

Supplementary Material 1

## Data Availability

The datasets generated and/or analysed during the current study are not publicly available due to the sensitive and confidential nature of the qualitative data involving vulnerable participants but may be available from the corresponding author on reasonable request and subject to ethical approval.
