# Supplementary material for: Ethical challenges in sensitive research: a reflective narrative on managing the clinician-researcher dual role
Source: BMC Palliat Care. 2025 Jul 19;24:205. doi: 10.1186/s12904-025-01850-y (PMC12275307; doi:10.1186/s12904-025-01850-y)
Supplement: Supplementary file 1 — Supplementary Material 1 [file 12904_2025_1850_MOESM1_ESM.docx]

**In-depth Interview Guide – Patients with Prostate Cancer**

**Title of Study**: Exploring Home-based Supportive and Palliative Care for men living with

Advanced-stage Prostate Cancer in a resource-limited Sub-Saharan African

Country (Ghana)*

**Participant group**: Men living with advanced prostate cancer

**Format**: Semi-structured interview with open-ended questions and follow-up probes

**Language**: English

**Section A: Introduction and Consent**

- Thank participant for agreeing to take part.
- Reiterate confidentiality, voluntary participation, and right to withdraw.
- Confirm verbal and written consent before beginning.
- Provide reassurance about emotional support and the option to pause or stop.

**Section B: Core Interview Questions with Probes**

1. Can you please walk me through your experience after receiving your prostate cancer diagnosis and how life has been since then?

*Probes*:

- How did you first find out about your diagnosis?
- What thoughts or feelings came to you at that time?
- How has your daily life changed since then?

1. How do you manage the condition at home, and who is involved in most of the care?

*Probes:*

- Are there specific tasks you or others perform regularly?
- Who helps you most with these tasks—family, friends, healthcare workers?

1. Could you tell me how the care is carried out at home? What role do you personally play in your own care?

*Probes:*

- Do you assist with medication, hygiene, wound care, or mobility?
- How do you feel about these responsibilities?

1. How do you get information about your condition and how to manage it?

*Probes:*

- Do healthcare workers explain things clearly?
- Do you look for information on your own (TV, radio, religious leaders, internet)?

1. How satisfied are you with the care you receive at home?

*Probes:*

- Are there aspects of care you think are good?
- Are there any aspects that disappoint you or cause frustration?

1. Please tell me more about the resources and support available for your care.

*Probes:*

- Are you receiving any emotional support (family, community, religious)?
- What about practical support (e.g. transportation, caregiving help)?
- Is financial support available from government, NGOs, or others?

1. Can you please share some common challenges or barriers you face with care at home?

*Probes:*

- Are there any difficulties with accessing medication or health professionals?
- Do you experience any stigma, embarrassment, or isolation?

1. In your own opinion, how can the care you receive at home be improved?

*Probes:*

- What kind of support would you like to have?
- Is there anything that could make you or your caregivers’ lives easier?

**Section C: Closing the Interview**

- Thank participant again for their time and openness.
- Ask if there’s anything else they’d like to share that wasn’t covered.
- Offer brief emotional support or refer to support systems as needed.

*This an abridged version of the interview guide could be found at <https://eprints.nottingham.ac.uk/56934/?template=etheses>

**Distress Management Protocol**

Given the sensitive nature of this study, emotional distress was a possibility. The following measures were in place:

1. Monitoring: Interviewer will observe for signs of distress (e.g., tears, long pauses, emotional withdrawal).
2. Pause/Stop Option: Participant will be reminded of their right to pause or stop the interview at any time without consequence.
3. Emotional First Aid: Interviewer will offer empathetic listening and allow time for the participant to recompose.
4. Referral: If distress is prolonged or intense, the participant will be referred to the hospital’s psychosocial support team or their healthcare provider with the participant’s permission.
5. Follow-Up: A follow-up call will be made within 24–48 hours to check on the participant’s well-being, if distress was observed or disclosed.
